# The Future of Medicine: How 3D Printing Is Transforming Pharmaceuticals

**DOI:** 10.3390/pharmaceutics17030390

**Published:** 2025-03-19

**Authors:** Jurga Bernatoniene, Jolita Stabrauskiene, Jurga Andreja Kazlauskaite, Urte Bernatonyte, Dalia Marija Kopustinskiene

**Affiliations:** 1 Department of Drug Technology and Social Pharmacy, Faculty of Pharmacy, Medical Academy, Lithuanian University of Health Sciences, Sukileliu pr. 13, LT-50161 Kaunas, Lithuania; jolita.stabrauskiene@lsmu.lt (J.S.); jurga.andreja.kazlauskaite@lsmu.lt (J.A.K.); urte.bernatonyte@stud.lsmu.lt (U.B.); 2 Institute of Pharmaceutical Technologies, Faculty of Pharmacy, Medical Academy, Lithuanian University of Health Sciences, Sukileliu pr. 13, LT-50161 Kaunas, Lithuania; daliamarija.kopustinskiene@lsmu.lt

**Keywords:** personalized medicine, 3D printing, innovative drug formulations, pharmaceutical manufacturing

## Abstract

Three-dimensional printing technology is transforming pharmaceutical manufacturing by shifting from conventional mass production to additive manufacturing, with a strong emphasis on personalized medicine. The integration of bioinks and AI-driven optimization is further enhancing this innovation, enabling drug production with precise dosages, tailored drug-release profiles, and unique multi-drug combinations that respond to individual patient needs. This advancement is significantly impacting healthcare by accelerating drug development, encouraging innovative pharmaceutical designs, and enhancing treatment efficacy. Traditional pharmaceutical manufacturing follows a one-size-fits-all approach, which often fails to meet the specific requirements of patients with unique medical conditions. In contrast, 3D printing, coupled with bioink formulations, allows for on-demand drug production, reducing dependency on large-scale manufacturing and storage. AI-powered design and process optimization further refine dosage forms, printability, and drug release mechanisms, ensuring precision and efficiency in drug manufacturing. These advancements have the potential to lower overall healthcare costs while improving patient adherence to medication regimens. This review explores the potential, challenges, and environmental benefits of 3D pharmaceutical printing, positioning it as a key driver of next-generation personalized medicine.

## 1. Introduction

The emerging 3D printing technology signifies a groundbreaking evolution in the production of medicines, transitioning from conventional technological methods to additive manufacturing. This advancement is particularly revolutionizing the pharmaceutical industry [1]. For example, in hospital settings, 3D printing will facilitate the creation of personalized drug dosages, helping patients with specific medical needs. Similarly, in community pharmacies, this technology will enable on-demand drug production, ensuring medications are tailored to individual patients, thereby reducing wait times and enhancing treatment outcomes [2].

Three-dimensional pharmaceutical printing is a pioneering advancement in personalized medicine, utilizing layer-by-layer deposition of pharmaceutical materials to produce customized medications with distinct structures, precise dosages, and tailored drug combinations [3]. This cutting-edge approach not only speeds up drug development but also drives innovation in pharmaceutical design, making it possible to create complex drug formulations that were previously hardly possible to produce [4]. Three-dimensional printing technology addresses one of the most pressing issues of healthcare: the move towards personalized treatment. Traditional mass production of medications often falls short, as the “one-size-fits-all” approach does not account for the unique requirements of each patient [5]. The rise of personalized medicine, powered by advancements in the understanding of genetics and lifestyles, highlights the need for more customized treatments [3,6,7]. Integrating 3D printing into healthcare settings revolutionizes clinical pharmacy by making drug manufacturing more accessible and adaptable to patient needs [8]. This not only enhances medication compliance and addresses logistical limitations but also has the potential to be cost-effective in the long run [9]. For instance, the on-demand production of drugs could reduce the need for large-scale manufacturing and storage, potentially lowering overall healthcare costs [10].

However, the widespread integration of 3D printing in the pharmaceutical industry faces numerous challenges that must be addressed for its successful adoption. Key obstacles include regulatory compliance, quality assurance, and engagement from healthcare professionals, all of which require urgent attention [11]. Establishing standardized guidelines for the production, safety, and efficacy of 3D-printed medications is essential to ensure consistency and reliability in patient care. The approval by FDA of the first 3D-printed drug marked a significant breakthrough, demonstrating the potential of additive manufacturing in pharmaceuticals. However, this milestone also highlights the ongoing need for clear regulatory frameworks that can keep pace with rapid technological advancements [12,13]. These frameworks must ensure patient safety, dosage precision, and product stability, while also facilitating innovation in drug design and production. Additionally, collaboration between regulatory agencies, pharmaceutical companies, and medical professionals will be critical in establishing best practices and gaining widespread acceptance of 3D-printed pharmaceuticals in clinical and commercial settings.

Our review will focus on innovative dosage forms enabled by 3D printing, underscoring the role of this technology in the future of pharmaceutical innovation. An overview of the current specifications, requirements, and production processes for customized 3D-printed pharmaceutical dosage forms will be provided, identifying the challenges and opportunities within the industry. It will also consider the potential environmental impact of 3D printing, such as reducing waste from unused or expired medications. Finally, this review will outline future directions and perspectives, based on the long-term impact of 3D printing on the pharmaceutical industry, healthcare delivery, and patient outcomes. This review will discuss the transformative potential of 3D printing in pharmaceuticals, offering insights into its current applications and future possibilities for advancing personalized medicine. Although there are many recent excellent reviews on the 3D printing of pharmaceuticals [2,4,9,13,14], our review explores in detail the advantages and disadvantages of 3D-printed pharmaceuticals, highlighting innovations that enable new tablet shapes, multilayered drug substances, and novel excipients. Our review emphasizes the role of 3D printing in developing personalized treatments for vulnerable groups like pediatric and geriatric patients or patients with chronic diseases and its unique ability to simplify complex drug regimens. Additionally, it examines innovative dosage forms made possible by 3D printing, discusses the future of the 3D technology for the printing of pharmaceuticals and provides an overview of the specifications, requirements, and production processes for customized 3D-printed drugs.

## 2. Pros and Cons of 3D Pharmaceutical Printing

**Figure 1:**
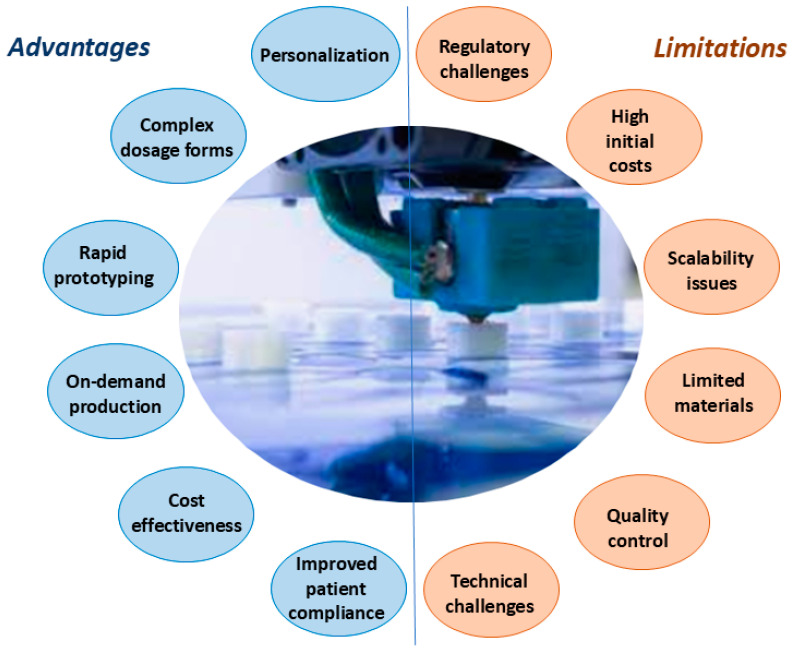
Advantages and limitations of 3D pharmaceutical printing.

The application of 3D printing in pharmaceuticals brings a range of benefits while also posing challenges, both of which play a crucial role in shaping its impact on drug development and patient care (Figure 1).

One of the most significant transformative advantages of 3D printing in the pharmaceutical industry is the ability to customize medication [9,13,15]. This technology enables the tailoring of drug combinations, release mechanisms, and dosages according to the specific requirements of each patient, thereby substantially improving treatment efficacy and adherence [14,16]. In addition to facilitating customization, 3D printing enables the creation of intricate structures and geometries that cannot be achieved using conventional manufacturing techniques, thus facilitating the advancement of innovative medication delivery systems [17,18,19]. Sophisticated drug release profiles, including staggered or delayed release, are made possible by these novel structures, which effectively optimize therapeutic outcomes while reducing adverse effects [9,15,17].

The research and development phases of the pharmaceutical industry can be accelerated due to the streamlined design and production of prototype medications made possible by the rapid prototyping capabilities of 3D printing [14,18,19]. Furthermore, the utilization of 3D printing technology enables hospitals and pharmacies to produce medications on demand [20,21]. This is particularly significant when it comes to the development of orphan pharmaceuticals or treatments for rare conditions, which are not feasible to produce on a large scale [20,21].

On-site manufacturing serves the dual purpose of reducing costs and waste related to drug overproduction and storage, while also accommodating the development of personalized medications that may have limited shelf lives [9,13,15]. In addition, the possibility to modify the dimensions, form, taste, or pigmentation of pharmaceutical substances renders them attractive and simpler to ingest, thereby enhancing adherence, especially among susceptible categories, including children and geriatric patients [22,23]. These unique properties make 3D printing a key player in advancing medication discovery and improving patient-centered care [13].

The integration of 3D printing in pharmaceuticals, while innovative, presents a range of challenges that slow down its broader applications [9,13,15]. Regulatory issues are a significant concern due to the personalized nature of 3D-printed drugs; each new formulation might necessitate a full approval process, which can be both costly and time-consuming [14]. The initial costs for setting up 3D printing technology are also substantial, encompassing expensive equipment, specialized training, and ongoing expenses such as software updates and maintenance [13,14,17].

In terms of scalability, 3D printing does not yet match the efficiency of traditional manufacturing methods for large-scale production, limiting its use to specialized drugs or niche markets [9,14,17,20]. Another obstacle is the limited range of materials suitable for 3D printing, as many active pharmaceutical ingredients and excipients used in traditional manufacturing are not compatible with current 3D printing technologies [9,13,14]. The relatively limited availability of excipients is the major obstacle for designing specialized dosage forms. Biodegradable, biocompatible, non-toxic, and stable excipients are essential for the wide application of 3D pharmaceutical printing [9,13,14]. Furthermore, with the increase in the more complex structures of dosage forms, continuous updating of modeling software for its design and production is necessary [14,15,17].

To avoid clogging or encourage consistency of the product, the control system, operating methods, and mechanical equipment must be updated and optimized [14,15,17]. The efficacy of the printed products could also be influenced by physicochemical parameters, including the surface tension and viscosity of the adhesives, as well as the properties of the nozzle of the 3D printer [14,15,17]. Furthermore, post-printing procedures such as drying methods, drying temperature, and drying time may influence the quality and appearance of the products [14,15,17].

Quality control also poses challenges; ensuring consistent quality and accurate dosages for each printed product is difficult, particularly since each printed product can have unique characteristics [13,15]. Moreover, due to the high precision required in the drug formulations, even small inaccuracies during the printing procedure may lead to incorrect dosages or release rates [14,18]. These factors require sophisticated quality control systems and contribute to the complexity of using 3D printing in pharmaceutical manufacturing. To ensure that the product satisfies all required safety standards, 3D printers used in the manufacturing of pharmaceutical formulations must also be validated [9,13,15].

Therefore, while the utilization of 3D printing in the pharmaceutical industry offers potential for advancements and customization, it requires thorough examination of its constraints and difficulties. Maintaining a meticulous balance among these parameters will be critical as this technology develops and becomes increasingly incorporated into traditional pharmaceutical manufacturing and healthcare environments.

## 3. Three-Dimensional Printing Techniques for Pharmaceuticals

The groundbreaking research on the innovative application of 3D printing technology in pharmaceuticals has brought to light its immense potential in creating a wide array of medicines. These studies have demonstrated that 3D printing can be used to develop rapidly dissolving orodispersible formulations [24], controlled release preparations [25], gastroretentive tablets [26], suppositories [27], minitablets [28], medical devices, and flexible multi-drug combinations [29]. This underscores the significant role of 3D printing in the future of pharmaceutical development.

**Figure 2:**
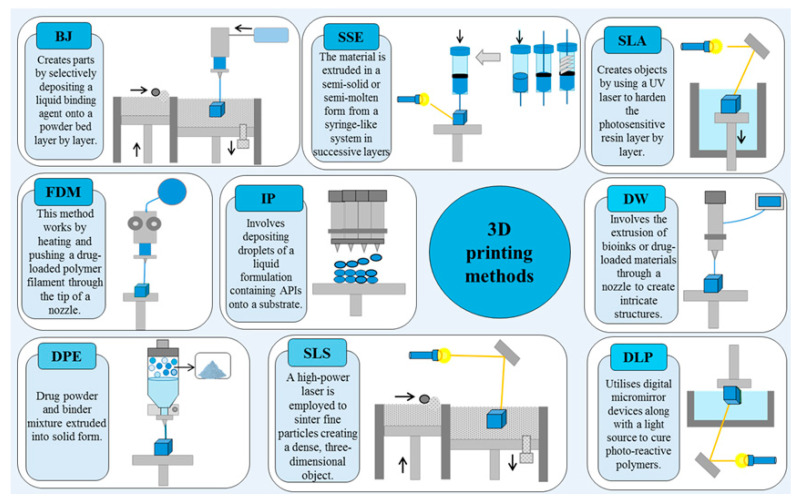
Overview of 3D printing methods for pharmaceuticals. BJ—binder jetting; SSE—semi-solid extrusion; SLA—stereolitography; FDM—fused deposition modelling; IP—inkjet printing; DIW—direct ink writing; DPE—direct powder extrusion; SLS—selective laser sintering; DLP—digital light processing.

A variety of 3D printing technologies (Figure 2) could be used in the pharmaceutical industry [9].

These include additive methods such as fused deposition modelling (FDM), direct powder extrusion (DPE), semi-solid extrusion (SSE), selective laser sintering (SLS), binder jetting (BJ), and stereolithography (SLA) (Figure 2). Other techniques, like digital light processing (DLP), inkjet printing, and direct ink writing (DIW), have also found their place in this innovative field (Figure 2). Each of these technologies contributes to the production of pharmaceutical products using 3D printing, showcasing the diverse applications of this technology.

FDM prints drugs by extruding hot melt polymers layer by layer, allowing for tailored drug release profiles [30]. DPE combines drug powders with binders to extrude and fuse them into solid dosage forms without solvents [31]. SSE extrudes semi-solid materials through a nozzle to create drug formulations that can contain hydrogels for controlled release [32]. SLS uses lasers to sinter powder materials, creating porous structures ideal for customized drug delivery mechanisms [33]. BJ-3DP deposits a liquid binder onto a powder bed to create complex multi-drug structures with different release kinetics. SLA uses UV light to harden photopolymer resin layers, producing high precision for complex drug delivery systems [34]. DLP rapidly cures photopolymer resins using a digital light projector, effectively creating detailed drug delivery devices. Inkjet printing precisely deposits droplets of drug solutions or binders to create complex multi-layered pharmaceutical products and directly deposits various materials onto a substrate, enabling the fabrication of complex, multifunctional pharmaceutical applications [35,36].

### 3.1. Fused Deposition Modelling (FDM)

FDM is highly regarded as one of the most widely researched and used 3D printing technologies in the pharmaceutical sector. Such significance is due to a relatively simple and economical setup, a wide range of materials used, and the ability to create complex dosage forms that improve patient compliance [37,38,39]. This method works by heating and pushing a drug-loaded polymer filament (which is initially prepared using hot melt extrusion) through the tip of a nozzle. The heated extrusion nozzle then moves along specific paths dictated by a computer-controlled design. The material is extruded layer by layer onto a build platform and then hardens when in contact with the build plate, forming the intended shape [30]. A major concern with this printing method is the potential degradation of drugs that can occur due to the high temperatures of the extrusion and printing processes; nevertheless, using the right excipients can achieve low-temperature printing [40,41].

FDM can process a diverse array of thermoplastic materials, each selected based on its specific mechanical properties and stability to suit various applications [42]. A critical factor in this process is the temperature setting. If the printing temperature is set too low, it can lead to the nozzle of the printer becoming blocked, which disrupts the continuity of the material extrusion and results in breaks along the printed paths. Conversely, setting the temperature too high can cause excessive softening of the plastic or, worse, lead to the degradation of the active pharmaceutical ingredient (API), compromising the quality and efficacy of the final product [24,40].

FDM typically operates at temperatures from 120 °C to 250 °C, which are ideal for processing polymers such as polyvinyl alcohol (PVA), polylactic acid (PLA), hydroxypropyl cellulose (HPC), and different types of Eudragit [43]. Studies investigating immediate release 3D-printed formulations, using PVP and Eudragit EPO, print at relatively lower temperatures in the range of 110–135 °C [41]. These materials are chosen because they extrude effectively at these temperatures. However, except for Eudragit E and HPC, most of these polymers are not optimal for creating immediate-release dosage forms [44].

FDM offers several significant advantages that make it attractive. This technology is notably user-friendly, with minimal setup and maintenance required, making it accessible to both beginners and experienced users. Compared to other 3D printing technologies, FDM equipment and materials are relatively inexpensive, reducing the entry barrier for small businesses and educational institutions [45]. As mentioned before, this method supports a diverse array of materials, each offering unique properties that can be customized to meet specific requirements [46].

However, FDM is not without its drawbacks. One of the primary limitations is its lower resolution and coarser surface finish when compared to other 3D printing technologies, such as SLA [47]. This can pose challenges in applications that demand high precision or smooth finishes. Additionally, parts produced via FDM can exhibit structural weaknesses at the layer junctions, making them vulnerable to bending and torsional stresses [45]. Although FDM allows for a broad selection of materials, the higher temperatures required to process advanced polymers may exceed the capabilities of standard FDM machines [40].

Hot melt extrusion (HME) is a preparatory part of FDM, involving mixing APIs and polymers, which are heated in an extruder until the mixture becomes a viscous molten state [48,49]. Critical parameters such as temperature and extrusion speed must be precisely controlled to protect the API from degradation and ensure the high resolution and stability of the final product. HME can be effectively combined with FDM to simplify manufacturing by eliminating the need for filament preparation. This combination has been successfully applied to both modified-release and immediate-release dosage forms [50,51]. Integrating HME and FDM into a single, continuous operation increases efficiency, automates production, reduces material waste, and facilitates the production of various dosage forms ready for immediate use [52].

FDM was used to produce 3D tablets containing the thermo-sensitive peptidomimetic drug enalapril maleate. The study assessed drug degradation during the printing process and explored methods to minimize it [53]. Also, the FDM technique allowed one research group to prepare 3D intragastric floating sustained release domperidone tablets [37]. Furthermore, another research group produced immediate-release 3D-printed tablets via FDM using the thermo-sensitive drug pantoprazole sodium. The study demonstrated the feasibility of FDM 3D printing for fabricating tablets with thermo-sensitive drugs [51]. Moreover, another study focused on low-temperature FDM 3D printing of thermolabile drugs such as ramipril [43]. This investigation highlighted the potential of FDM to fabricate immediate-release tablets containing heat-sensitive active pharmaceutical ingredients without significant degradation [43]. Moreover, the development of personalized, rapidly dissolving captopril tablets using FDM 3D printing was successfully demonstrated, offering a promising solution to address individual patient needs [54].

### 3.2. Direct Powder Extrusion (DPE)

Direct Powder Extrusion (DPE) is a 3D pharmaceutical printing technique that enables the direct processing of powder-based drug formulations into customized dosage forms without requiring solvents or extensive post-processing. DPE applies heat, pressure, or mechanical force to convert dry powder blends into a semi-molten state, allowing for controlled extrusion through a nozzle to form structured drug products. One of the key advantages of DPE is its ability to produce high drug-load formulations, making it suitable for applications where potent APIs are required in precise doses [55]. Additionally, since the process does not rely on solvents, it eliminates drying steps, reduces manufacturing complexity, and minimizes the risk of solvent-related stability issues. DPE also supports complex geometries and multi-layer drug delivery systems, enabling tailored drug release profiles such as sustained, immediate, or pulsatile release formulations. DPE directly forms drug-loaded prints from powder, skipping the intermediate steps seen in other 3D printing methods, and operates at lower temperatures, making it ideal for heat-sensitive materials. However, this generally results in a less uniform distribution of the API than HME, which can affect the quality of the final product [55].

Researchers successfully fabricated paracetamol tablets using DPE, demonstrating the capability of this technique to produce immediate-release tablets with uniform drug distribution and desired mechanical properties [56]. A study utilized DPE to create sustained-release tablets of theophylline. The research highlighted the method’s potential in tailoring drug release profiles by adjusting formulation parameters [57]. Another study developed and validated a DPE-based 3D printing method for rapidly dissolving Levodopa/Carbidopa tablets, suitable for integration into hospital pharmacies. Levodopa was printed successfully; however, Carbidopa was incompatible with the printing process, highlighting challenges in formulating certain drugs using this technology [58].

### 3.3. Semi-Solid Extrusion (SSE)

SSE is a printing technique that involves extruding material in a semi-solid or semi-molten form from a syringe-like system in successive layers to form a three-dimensional object. This method is particularly versatile in pharmaceutical manufacturing, allowing for the creation of complex drug formulations that are impossible with traditional extrusion techniques. The initial substances, typically gels or pastes, are created by blending the different proportions of various materials with solvents to achieve a viscosity that is ideal for printing [59]. SSE has been utilized in developing various dosage forms such as immediate-release tablets, orodispersible tablets, pediatric chewable gummies, controlled-release tablets, gastrofloating tablets, and solid lipid tablets [60].

The advantages of SSE compared to solid extrusion is its ability to process a broader range of materials, including those that are temperature sensitive. This is because SSE operates at lower temperatures, preserving the integrity and effectiveness of active ingredients, which would be compromised by the heat needed to melt solids [61]. SSE is also more efficient and generates less waste than conventional extrusion techniques. It extrudes materials in a semi-solid state, reducing the need for extensive heating and post-processing, lowering energy consumption and minimizing material waste.

However, SSE also presents some challenges. The excipients used must be carefully chosen for compatibility and must possess the right viscosity and rheological properties to support effective printing. Issues such as too low a viscosity can cause uncontrollable material flow, whereas too high a viscosity might impede the flow entirely. Moreover, post-processing steps like drying or cooling are often necessary, and the physical state of the initial material can influence these processes, sometimes resulting in shrinkage, deformation, or even collapse of the structure if it lacks sufficient hardness. Additionally, SSE generally produces lower-resolution prints because the printer’s die heads must be large enough to handle the high viscosity of the materials, unlike the finer extrusion orifices used in other printing methods [60,62].

### 3.4. Selective Laser Sintering (SLS)

SLS is a 3D printing method that employs a high-power laser to sinter fine particles of plastic, metal, ceramic, or glass, creating a dense, three-dimensional object. The process begins with a thin layer of powder spread over the surface of a built platform. A laser then selectively sinters the powder, tracing a cross-section of the part onto the powder bed. After each layer is fused, the build platform lowers, and a new layer of powder is applied on top. This process is repeated until the entire object is completed [63]. SLS technology eliminates the need for support structures because the powder around naturally supports the object throughout the printing process. This advantage enables the production of complex designs that are challenging with conventional manufacturing methods. Unlike processes such as FDM, which require the preparation of filaments, or BJ and SSE, which necessitate post-processing like drying, SLS is a straightforward, single-step method. The SLS technique is characterized by extraordinary precision. The powder dispenser and building platform feature a 0.1 µm resolution, enabling precise control over layer thickness for sub-micrometer precision. This high level of accuracy is vital for producing components with strict tolerances [64]. This high level of detail is especially beneficial for pharmaceutical uses, where the geometric design of a dosage form can greatly influence its effectiveness and the pattern of drug release. SLS is adept at creating advanced drug delivery systems, including multi-layer tablets that incorporate various APIs or excipients, each engineered to dissolve at predetermined intervals. Fina et al. created tablets with a cylindrical, gyroid lattice and bi-layer structures that influenced release characteristics [65].

Scaling the SLS process presents several challenges, primarily due to the large investment in equipment and powder management systems to ensure consistent quality and efficiency at higher production volumes. The cost of laser sintering machines and the need for the highest quality powder often hinder the transition from prototype to full-scale production. In addition, the use of SLS in the pharmaceutical sector has been somewhat limited because the intense laser energy can cause drug degradation [66].

Researchers employed SLS to produce carvedilol tablets with various dosages (3.125, 6.25, and 12.5 mg). The study demonstrated the capability of SLS to fabricate personalized dosage forms meeting pharmacopeia standards in terms of strength, friability, active dose, and dissolution rate [67]. Another study explored the feasibility of using SLS with a CO_2_ laser to produce solid oral dosage forms containing copovidone and paracetamol without the need for absorbance enhancers [68]. The results indicated that successful sintering depends primarily on powder flowability influenced by particle size, while low compactness limited mechanical strength, demonstrating SLS as a promising technique for personalized medicine formulations [68].

### 3.5. Binder Jetting (BJ)

BJ is a versatile additive manufacturing technology that creates parts by selectively depositing a liquid binding agent (which is typically a polymeric liquid) onto a powder bed layer by layer. This process is similar to how a standard inkjet printer works, but a binding agent is used instead of ink. After each layer is printed, the platform lowers, a new layer of powder is added, and the process repeats until the part is complete. Once printed, any unbound powder is cleared away to reveal the item. These items are generally fragile and usually need extra processing, such as baking in an oven or soaking with other materials, to improve their strength and functionality [69].

Although BJ can produce high-resolution parts with layers as small as 50 µm, its resolution is limited by the size of the binder droplets and the powder’s granularity. Compared to methods such as SLA or SLS, the surface finish of BJ-printed parts is less smooth and often requires further processing to achieve the desired smoothness for a specific application. In addition, the accuracy of BJ is affected by the powder dispersion method and the material penetration stage, both of which can affect the final product’s dimensional accuracy and structural integrity [69,70].

In the literature, BJ technology has been applied to develop various intricate pharmaceutical formulations, including controlled-release tablets, fast-dissolving tablets, and delayed-release tablets [71,72]. The most notable and initially FDA-approved product utilizing this technology is Spritam by Aprecia Pharmaceuticals. Aprecia Pharmaceuticals modified the binder jetting approach to establish a continuous production-like process. This innovation allows for the large-scale production of oral dosage forms through a singular, streamlined operation known as ZipDose^®^ technology (Aprecia Pharmaceuticals, Mason, OH, USA) [72].

### 3.6. Stereolithography (SLA)

SLA is an additive manufacturing form of 3D printing technology that creates objects by using a UV laser to harden the photosensitive resin layer by layer. In the medical and pharmaceutical sectors, SLA is primarily employed for tissue engineering, making implantable devices, and printing tablets that incorporate multiple drugs, as well as fabricating soft devices that release drugs [73,74]. Despite these uses, its application in pharmaceuticals remains limited. A significant limitation is the scarcity of photocrosslinkable polymers approved for medical use, coupled with potential safety concerns such as the carcinogenicity of some resins, which warrants further investigation. Additionally, the photosensitive nature of the materials used may compromise the long-term stability of drug formulations [60].

SLA offers considerable benefits. It particularly stands out due to its precision capabilities which are crucial for creating detailed and accurate models. This high level of detail ensures that components such as microfluidic devices, which are often used in biomedical applications for handling liquids at very small scales, are manufactured with exacting precision. The affordability and compact size of SLA machines also offer substantial advantages. These machines are more cost-effective not only in terms of initial investment but also in terms of running costs, as they consume less power and use less expensive materials compared to SLS systems. Furthermore, the ability to monitor the printing process in SLA machines through clear enclosures enhances quality control, allowing technicians to make adjustments in real time. This is especially beneficial in pharmaceutical applications where even minor imperfections can render a part unusable. SLA also reduces local heating during printing, which preserves the integrity of heat-sensitive drugs in oral dosage forms [34,75].

Researchers used SLA to create implants incorporating 0.5% levofloxacin, an antibiotic, into a flexible resin. The study demonstrated that the implants maintained mechanical integrity and exhibited high antimicrobial activity, suggesting potential for localized infection treatments [76]. SLA was also employed to fabricate intravesical devices designed for the controlled release of lidocaine hydrochloride, a local anesthetic [77]. These devices aim to provide targeted therapy for bladder conditions, enhancing treatment efficacy and patient comfort [77].

All 3D printing techniques are overviewed in Table 1.

### 3.7. Additional 3D Printing Techniques

Three-dimensional printing technologies are evolving rapidly, expanding the possibilities for their application in the pharmaceutical industry. Beyond the previously discussed methods, there are other significant techniques, such as digital light processing (DLP), inkjet printing (IP), and direct writing (DW). Each offers unique capabilities in terms of material compatibility, resolution, and potential for pharmaceutical innovation.

#### 3.7.1. Digital Light Processing (DLP)

DLP utilizes digital micromirror devices (DMD) along with a light source to cure photo-reactive polymers. This process is similar to SLA but typically offers faster speeds because it cures entire layers simultaneously [80]. DLP can produce highly precise and smooth drug delivery devices, such as microneedle arrays for transdermal drug delivery. Its ability to quickly create complex structures makes it suitable for high-throughput fabrication of dosage forms that are tailored to individual patient needs [81].

Compared to SLA, DLP can achieve similar or faster production times and often results in smoother finishes due to the nature of the layer curing process. It offers a similar resolution and material range but excels in faster prototype turnaround, making it ideal for iterative development processes in drug formulation [82].

Researchers used a one-step DLP 3D printing technique for creating silk fibroin-based microneedles from aqueous protein solutions at low concentrations. The resulting microneedles demonstrated flexibility and successful skin delivery capabilities, validated using rhodamine B fluorescent dye [83].

#### 3.7.2. Inkjet Printing (IP)

IP in pharmaceuticals involves depositing droplets of a liquid formulation containing APIs onto a substrate. This method has great flexibility in terms of substrate, as formulated inks can be printed onto a wide variety of materials, including films, microneedles, clinical stents, contact lenses, and even nails [84]. This method is particularly effective for creating personalized medicines as it allows for precise control over the dosage and placement of multiple APIs within a single tablet. Inkjet printing is also being explored to print biosensors directly onto drug forms, enabling integrated drug delivery and monitoring systems [85,86].

While inkjet printing offers lower resolution compared to DLP or SLA, its flexibility in handling a variety of liquid materials and active ingredients without the need for high temperatures makes it particularly valuable. It stands out in material compatibility, especially for producing complex multi-drug formulations and devices with embedded electronic functionalities [87].

Researchers evaluated thermal IP as a rapid method for accurately depositing personalized drug doses onto oral films. Using a modified HP cartridge, aqueous solutions of salbutamol sulphate were successfully printed onto potato starch films [88]. IP has been also used for precise dose control of warfarin onto orodispersible films to improve patient-specific dosing [89].

#### 3.7.3. Direct Writing (DW)

DW comprises a variety of process technologies that are multifaceted, adaptable, and function across multiple scales. This technique is also known as direct printing or digital writing. DW involves the extrusion of bioinks or drug-loaded materials through a nozzle to create intricate structures. It can handle a wide range of viscosities and often incorporates elements of bioprinting, using cells and biocompatible materials [90].

This method is a powerful tool in advanced manufacturing, particularly useful in the field of regenerative medicine and for fabricating drug delivery systems, such as implants or scaffolds with controlled release properties. Unlike traditional 3D printing methods, DW offers exceptional material versatility and can process “living” inks. This capability is crucial for directly writing cells and biomaterials, making it pivotal for developments in medical treatments and therapies [90,91,92]. However, its resolution may be inferior to that of DLP or SLA but superior in terms of potential for creating biologically active structures and complex geometries integrated with live cells or growth factors.

## 4. Bioinks, New Tablet Shapes, and Different Layers of Drug Substances

**Figure 3:**
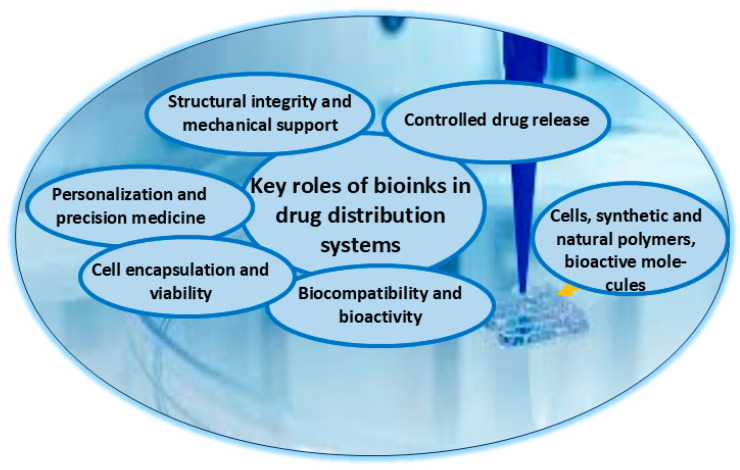
Specific roles of bioinks in drug distribution [93,94,95].

Bioinks are specialized biomaterial formulations used in 3D pharmaceutical printing to create customized drug delivery systems, implantable scaffolds, and bioprinted tissues [93,94,95]. These inks must be biocompatible, printable, and capable of encapsulating active pharmaceutical ingredients while ensuring stability and controlled drug release (Figure 3).

The use of bioinks has revolutionized personalized medicine, allowing for the fabrication of patient-specific drug formulations with precise dosages and tailored release profiles. Bioinks are composed of natural and synthetic polymers such as alginate, gelatin, polylactic-co-glycolic acid, and polyethylene glycol, which provide structural support and drug-carrying capabilities [93,94,95]. Hydrogels, including gelatin-methacrylate and alginate-based gels, create a hydrated environment suitable for sustained drug release [93]. The incorporation of active pharmaceutical ingredients, such as antibiotics, chemotherapy agents, and pain relievers, enables targeted and controlled therapy [96,97,98]. Crosslinking agents enhance mechanical integrity, while nanoparticles and microspheres could improve drug stability and release kinetics [99]. The versatility of bioinks in 3D pharmaceutical printing allows for the development of personalized drug delivery formulations which facilitate sustained and controlled drug release, reducing dosing frequency and improving treatment compliance [96]. Bioprinted scaffolds can be implanted for localized drug delivery, particularly in post-surgical pain management or tissue regeneration [100,101,102]. Additionally, bioinks combined with living cells enable bioprinted tissues for applications in wound healing and regenerative medicine [103,104]. Antibacterial and bioactive components can also be incorporated into wound dressings to accelerate healing and prevent infections [105]. Despite its promise, bioink-based 3D pharmaceutical printing faces challenges, including the stability of active drugs during printing, scalability of production, regulatory approval, and standardization of formulations [106]. The future of bioink technology lies in AI-driven optimization, smart hydrogels, and multi-material printing, which will further refine drug delivery precision and enhance patient outcomes [107,108]. As technology advances, bioinks are set to play an important role in the next generation of personalized and on-demand medicine.

Integrating 3D printing into pharmaceutical technology marks a transformative evolution in the shapes and compositions of tablets, shifting away from traditional drug manufacturing methods [4,109]. Traditionally, tablets are formed through compression and may be coated to provide a smooth exterior layer or left uncoated. Meanwhile, capsules are produced by encapsulating medicinal and dry ingredients around a core material [110,111,112]. The bioavailability and release characteristics of these dosage forms can differ significantly. The specific therapeutic application of an oral solid dosage product dictates whether its release will be immediate, sustained, controlled, or extended. These release properties are crucial in selecting the appropriate drug manufacturing methods and the equipment and technologies used in production [111]. The remarkable flexibility of digital design and the precision of printing technology have greatly enhanced the capabilities of 3D printing in this field, enabling the creation of tablets with intricate geometries, dual-drug, bilayer, and multi-layer tablets.

**Figure 4:**
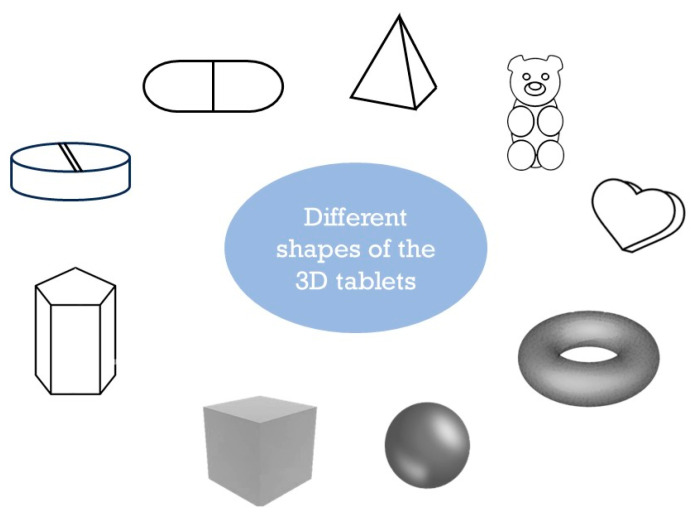
Examples of various tablet shapes created using 3D printing methods.

Traditionally, pharmaceutical tablets have been limited to conventional shapes, such as round, oval, or rectangular [110]. Three-dimensional printing has enabled the creation of tablets with complex geometries, such as spirals and hollow structures (Figure 4).

These designs can modulate the surface area in a way that affects the dissolution rates, thus controlling the release profile of the drug. Complex tablet structures are another area where 3D printing shines, allowing for creating tablets with specific geometries that control drug release rates. This precision is crucial for diseases like Parkinson’s, where controlled drug release enhances treatment stability and patient compliance [113]. Research by Xu et al. examined three tablet models (cylinder, horn, and reversed horn) with controlled structures, finding that drug release rates vary based on internal tablet architectures. The cylinder model maintained a steady drug concentration, the horn model increased drug release suitable for patients with developing drug resistance, and the reversed horn model showed a decreasing release profile, beneficial for treating hypertension [114]. Goyanes et al. also explored how geometry affects drug release kinetics, successfully 3D-printing tablets in five shapes: cube, pyramid, cylinder, sphere, and torus. Drug stability remained intact, with release rates determined by the surface area to volume ratio, underscoring the influence of shape. Drug release was regulated through an erosion-mediated process [115]. Another study focused on the fabrication of mini-tablets with polyhedral shapes, from tetrahedron to icosahedron, using an FDM 3D printer to investigate the impact of geometric shape on drug release kinetics, confirming that 3D printing enables precise control over drug dosage by adjusting the shape of the drug while maintaining a controlled release profile, highlighting its potential for personalized and pediatric formulations [116]. Researchers studied the development of innovative low-dose hydrocortisone oral forms for pediatric use through hot melt extrusion and fused deposition modeling 3D printing, where mini-waffle-shaped tablets containing 2, 5, and 8 mg of the drug were successfully printed, offering a pediatric-friendly alternative to compounded capsules while ensuring uniformity, stability, and rapid drug release similar to conventional forms [117].

Dual-drug tablets are engineered to contain two or more distinct APIs in separate compartments, thus preventing interactions between the components while offering combined therapy advantages. These tablets are especially useful in treating diseases such as HIV/AIDS and cancer, where multiple medications are typically prescribed to manage the condition effectively [118]. The use of 3D printing technology facilitates precise dosing within a single tablet, which minimizes the number of pills patients need to take and simplifies their treatment plans. In research by Lee et al., antibiotic-loaded biodegradable polymer scaffolds were developed using 3D printing, followed by encapsulation with a hydrogel containing a second antibiotic aimed at treating chronic osteomyelitis. Although not entirely produced by 3D printing, this method proved to be effective [119]. In another study, McDonagh et al. demonstrated that altering tablet designs makes it possible to create dual-drug tablets that release drugs simultaneously, delay release, or allow for pulsatile release. This innovation facilitates the development of complex, multi-API release profiles that enhance the effectiveness of personalized medications [120].

Multi-layer tablets can contain several layers, each with a different drug or different release characteristics. These tablets made through 3D printing can house separate layers of medication. For instance, a bilayer tablet might combine an immediate-release layer with a slow-release layer to provide both rapid and prolonged effects [121]. These tablets can be particularly beneficial for chronic conditions such as bipolar disorder [122] or complex cardiovascular diseases [123], where different medication is needed, and the release can mimic the body’s natural rhythms or provide a sequential therapeutic effect.

Tabriz et al. developed 3D bilayer tablets utilizing dual-controlled release for treating tuberculosis. The team formulated the tablets with two separate layers, each containing a different drug: isoniazid for release in the acidic stomach environment and rifampicin for release in the alkaline upper intestine. They successfully produced these bilayer tablets using FDM technology [124]. The use of this technology represents a significant advancement in the development of personalized medicine. Robles-Martinez et al. used SLA technology to incorporate six drugs in one multi-layered tablet. Even though this study needs more research to achieve optimal dosing and dimensions, it demonstrated acceptable physicochemical characteristics and differing drug release profiles [73]. The use of 3D printing has also successfully produced bilayer tablets with two model drugs for tuberculosis treatment. These tablets had covering layers that were carefully designed to achieve a fast release of isoniazid and delayed release of rifampicin [124]. The integration of artificial intelligence (AI) and 3D printing technology enabled the precise design and fabrication of customized drug capsules, optimizing drug release through structural modifications rather than formulation changes in the study of Hu et al. [125]. Using a genetic algorithm and fused deposition modeling (FDM), 3D printing multi-layer capsules containing isoniazid and acetaminophen have been created, ensuring targeted drug release profiles with high accuracy and reliability, which could advance the development of personalized oral dosage forms [125].

## 5. Innovative Substances for 3D-Printed Pharmaceutical Forms in Geriatric and Pediatric Applications

Three-dimensional (3D) printing is revolutionizing the pharmaceutical industry and clinical pharmacy, shifting away from traditional mass production toward the creation of customized, patient-specific medications [126]. This technology enables the personalization of oral dosage forms, allowing for precise control over size, shape, drug release profiles, and dosage modifications [60], making it particularly beneficial for populations with specialized medical needs, such as pediatric and geriatric patients.

The integration of 3D printing in drug formulation plays a crucial role in improving medication adherence and therapeutic efficacy in these vulnerable groups. Pediatric patients often require chewable, flavored, or rapidly dissolving tablets to enhance palatability and ease of administration, while geriatric patients benefit from instant-dissolving tablets (IDTs) that reduce swallowing difficulties and improve bioavailability [28,127,128]. Additionally, novel excipients, specifically designed for 3D printing, emerge as key components in formulating age-appropriate drug products, ensuring both structural integrity and controlled release mechanisms.

**Figure 5:**
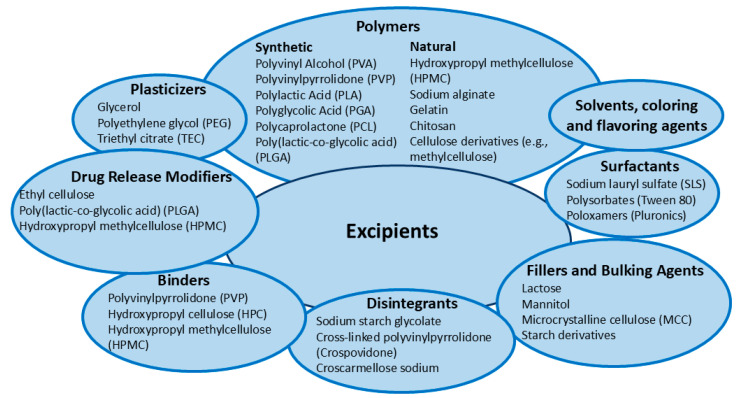
The main excipients used in 3D drug printing [31,46,84,109,129,130].

When selecting excipients or materials for 3D drug printing, key considerations include the desired drug release profile (immediate versus sustained release), the specific printing method used (such as extrusion, inkjet, or selective laser sintering), and the stability and compatibility of excipients with active pharmaceutical ingredients [31,46,84,109,129,130]. Additionally, materials must promote patient acceptance and compliance by ensuring suitable palatability and ease of administration, while also satisfying regulatory requirements regarding biocompatibility and toxicity (Figure 5).

Polymers are widely used in 3D drug printing due to their flexibility, printability, biocompatibility, and ability to control drug release and stabilize APIs. Plasticizers further enhance flexibility and processing characteristics, while fillers and bulking agents provide structural stability and improve mechanical properties. Disintegrants ensure rapid breakdown after administration, and binders maintain the integrity of printed dosage forms. Surfactants improve wetting, dissolution, and dispersion of APIs, while drug release modifiers control or modify the drug release profiles. Solvents are crucial for inkjet or extrusion-based printing methods, and colorants and flavoring agents enhance dosage form appearance, patient compliance, and palatability [31,46,84,109,129,130] (Figure 5).

**Figure 6:**
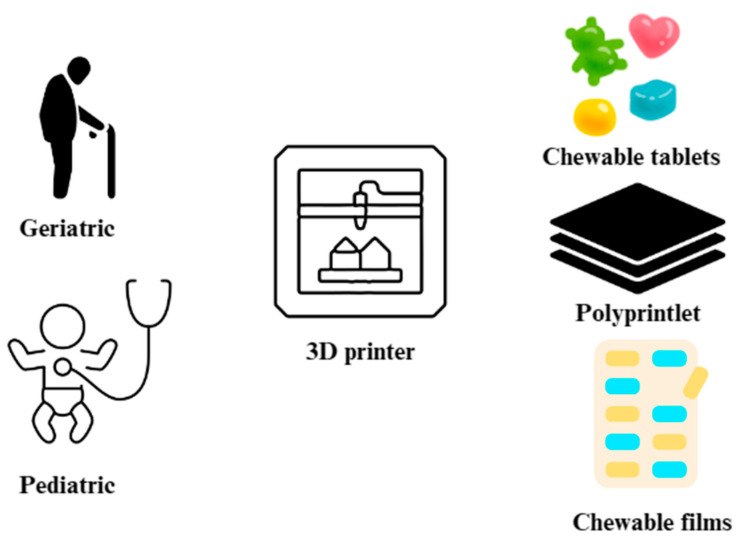
The most used 3D pharmaceutical forms for geriatric and pediatric patients.

Three-dimensional printing enables the production of both solid and semi-solid dosage forms. Although there have been great advances in the various routes of drug administration, the oral route is still considered the most convenient and is preferred by a large number of consumers worldwide [60]. The following dosage forms can be printed with the 3D printer: tablets, capsules, pellets, polypills, gastroretentive delivery systems or oral films. By far the most popular dosage forms among geriatric, pediatric and dysphagia patients are chewable tablets. They are oral medications prepared to be chewed before swallowing to facilitate easier intake or ensure proper release of the active ingredient [7]. Chewable tablets are recognized for their smooth texture, pleasant taste, and absence of residual bitterness. These tablets offer standard tablet production and dosing benefits while being more patient friendly. For pediatric patients, 3D printing allows the printing of a dosage form containing a small amount of the active substance, so that the patient does not have to manually divide the tablet by hand. This process often leads to uneven weight distribution after division, and it can also cause drug release problems. Swallowing tablets or capsules can be problematic for the dysphagia patient group, requiring modification of the formulation (e.g., crushing tablets or opening capsules) to facilitate administration [131]. The uncomfortable process of swallowing the tablets may deter older patients from taking their medicines—in this situation chewable tablets are a great choice. Moreover, chewable tablets are not constrained by size, as they are designed to be chewed before they are swallowed [132]. However, there are some disadvantages regarding this dosage form. Some medicines may be unpalatable, making it difficult to insert them into a chewable tablet without causing discomfort to the patient during administration. For that reason, sweeteners and flavoring agents are used, often in large amounts. Hardness is another important factor to consider. Chewable tablets have been reportedly associated with incidents involving tooth damage or denture breakage [131]. Excipients play an important role in ensuring the safety and efficacy of this formulation. Typically, when formulating a medicine, the choice of excipients will depend on several factors, such as the type of dosage form, the production method, the API properties, and the intended drug release profile [131]. The most used 3D pharmaceutical forms for geriatric and pediatric patients are shown in Figure 6 [133,134].

SSE 3D printing technology is often chosen for printing chewable tablets. This printing method is affordable, simple, and suitable for printing thermolabile drugs. Lower printing temperatures can be employed when using the SSE method compared to FDM. Excipients which are used for manufacturing chewable tablets using conventional methods, can also be used for the preparation of 3D-printed chewable formulations [131]. Excipients that are used in the preparation of chewable tablets can be divided into these categories: sweeteners, flavoring agents, colorants, and gelling agents. The excipient belonging to each group performs a specific function in the chewable tablet formulation. Some examples of sweeteners are mannitol, sorbitol, and dextrose. The function of sweeteners is to provide the necessary properties of sweetness and chewability [135]. Flavoring agents are usually fruit-based substances which give the medicine a strawberry, banana, or grape taste and mask the unpleasant taste of the APIs. Gelling agents such as cellulose derivatives, starch or gelatin are used to modify the viscosity and texture of the tablets. Diluents and pH modulators play an important role in the chewable tablet’s formulation. pH modulators (e.g., citric acid) ensure proper pH of the formulation (about pH 3–5). Diluents are used to increase weight and improve the content uniformity of the tablet. Polyols are examples of diluents used in chewable tablets.

However, 3D printing requires the application of innovative substances (Table 2) to ensure therapeutic efficacy of the drug formulation [131,135].

The lipid Gelucire 50/13 is an example of the main excipient for the development of the pharma-ink due to its favorable printability and its prior use in SSE 3DP [136]. Lipid excipients in 3D printing have advantages that are important in the printing process such as low melting temperature and rapid recrystallization. Gelucire^®^ 48/16 is another example of an excipient for lipid-based formulations. This non-ionic surfactant has solubilizing properties that increase bioavailability of poorly soluble API. Carrageenan is a polysaccharide with thermal responsiveness and gelling ability that can improve the thermal stability and mechanical strength of the gel ink when combined with gelatin [137]. Bitter chocolate is used as palatability enhancer in chewable tablets designed for pediatric patients. This excipient has antioxidant properties and has a positive effect on cognitive function. It can be mixed with corn (glucose) syrup. Corn (glucose syrup) is used to facilitate the incorporation of both hydrophilic and lipophilic APIs into the chewable tablet. Although starch is commonly used as an excipient in the pharmaceutical industry, it is important to note that starch from different botanical origins can lead to different drug release kinetics [132].

**Figure 7:**
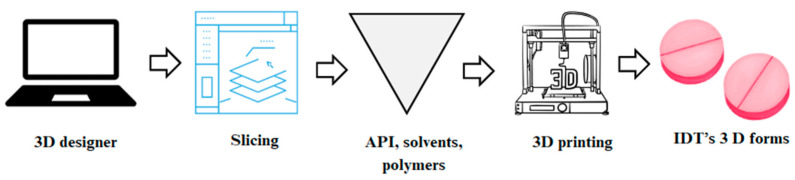
The workflow for producing IDT 3D-printed drugs, from digital design and preparation to the actual 3D printing and the final medication form.

Three-dimensional technology allows the production of dissolving or instant-dissolving tablets (IDTs) with a complex porous structure that dissolves quickly in the mouth or water (Figure 7).

The higher the porosity, the quicker the disintegration. This technology is particularly beneficial for patients who have difficulty swallowing tablets.

Three-dimensional printing is also used for orally disintegrating tablets (ODTs). ODTs are oral solid dosage forms that disperse almost instantly in the mouth and can be swallowed without needing water co-administration [138]. ODTs are especially beneficial for psychiatric, geriatric, pediatric patients, or patients with dysphagia. Rapid disintegration makes the swallowing process easier for these patient groups. Since both ODTs and IDTs can be molded into different shapes, they are ideal for children who find them visually appealing [139]. Disintegrating agents play a crucial role in allowing tablets to divide into smaller parts when in contact with an aqueous solution [140]. Superdisintegrating agents are used in the formation of FDTs (fast dissolving tablets) for their excellent disintegrating qualities at a relatively low concentration [141]. Examples include croscarmellose, crospovidone, SSG, and hydroxypropyl cellulose (HPC) [140]. Croscarmellose sodium is a pharmaceutical excipient and one of the most frequently used disintegrants in oral pharmaceuticals, both in capsule and tablet formulations. Crospovidone and sodium starch glycolate (SSG) are also used as disintegrants due to their porosity. Water flows due to capillarity inside the tablet and causes swelling, rupture, and disintegration. HPC tablets begin gelling immediately on contact with water, creating a cellulosic matrix. HPMC is used as a binder, but it has some advantages, such as dissolvability and low toxicity [142]. PEG is a water-soluble and biodegradable excipient. It can be used together with naproxen by preparing dispersion. PVA can improve the mechanical strength and drug release properties of the printed dosage forms. PVA has sufficient thermoplastic properties to be printed using the fused deposition modeling (FDM) technique. PVA can be mixed with a plasticizer to prepare the filament for printing [14].

Sublingual administration is a method where medication is placed under the tongue for absorption, providing a quick onset of action and higher bioavailability than oral intake. It primarily benefits children and individuals with swallowing difficulties. Dysphagia is a common problem of all age groups. The elderly, children, and patients who are mentally retarded, uncooperative, nauseated, or on reduced liquid intake/diets especially have difficulties in swallowing these dosage forms [143]. Sublingual administration of medicinal substances includes nitroglycerin, buprenorphine, zolpidem, ergotamine, asenapine, etc. For this reason, 3D-printed sublingual tablets are beneficial for cardiovascular and psychiatric patients and for patients with insomnia. Three-dimensional printing can be used to print sublingual tablets that have the exact amount of API that is needed for the particular patient. Excipients used in sublingual formulations include lactose, dextrose, sucrose, and mannitol (Table 3).

Mixtures of these ingredients can be used to ensure quick dissolution of the tablet. Lactose can enhance the compatibility, physical stability, and drug solubility of the powder mixture used in 3D-printed tablets [14]. Kollidon enables the instant release matrix of the formulated tablet; it is a great solubilizer that dissolves in all hydrophilic solvents. PEARLITOL^®^ 50 C is used as a filler and bulk sweetener; this excipient has great stability and no hygroscopicity. Compressol SM is a directly compressible excipient that consists of mannitol and sorbitol. Mannitol provides quick binding and disintegration. Sorbitol is used as plasticizer, stabilizing, and sweetening agent, and tablet diluent. The function of excipients in the development of sublingual formulations is to ensure the rapid relaxation of the active substance after administration.

Thus, the development of ODTs and IDTs and sublingual pharmaceutical forms relies on excipients that enhance rapid disintegration, solubility, and absorption. Superdisintegrants like crospovidone and croscarmellose sodium facilitate a quick breakdown, while effervescent agents (citric acid, sodium bicarbonate) accelerate dispersion. Fillers and sweeteners such as mannitol and xylitol improve mouthfeel, and film-forming polymers maintain tablet integrity while ensuring fast dissolution [140]. For sublingual forms, mucoadhesive polymers like carbomers and sodium alginate prolong retention and enhance bioavailability. These excipients optimize patient compliance and drug efficacy, particularly for pediatric, geriatric, and emergency use.

## 6. Personalized Treatment and Complex Drug Regimens for Pediatric and Geriatric Patients

Personalized medicine represents a revolutionary approach in healthcare, crucial for managing complex drug regimens tailored to individual patient profiles. This approach is particularly important for pediatric and geriatric populations, who often require complex medication regimens due to age-specific physiological differences, varying metabolic rates, and distinct therapeutic requirements [154]. In pediatric patients, dosage precision, palatability, and ease of administration are key considerations, while geriatric patients often face challenges related to polypharmacy, altered drug metabolism, and swallowing difficulties [155,156,157]. Advanced drug delivery technologies, such as 3D-printed pharmaceuticals and innovative excipient formulations, enable the customization of medications in terms of dosage, release profiles, and administration routes, ensuring optimal efficacy and safety for these vulnerable groups. While traditional drug manufacturing methods are currently used to achieve non-conventional dosing, it is important to note their regulation and quality control limitations.

**Figure 8:**
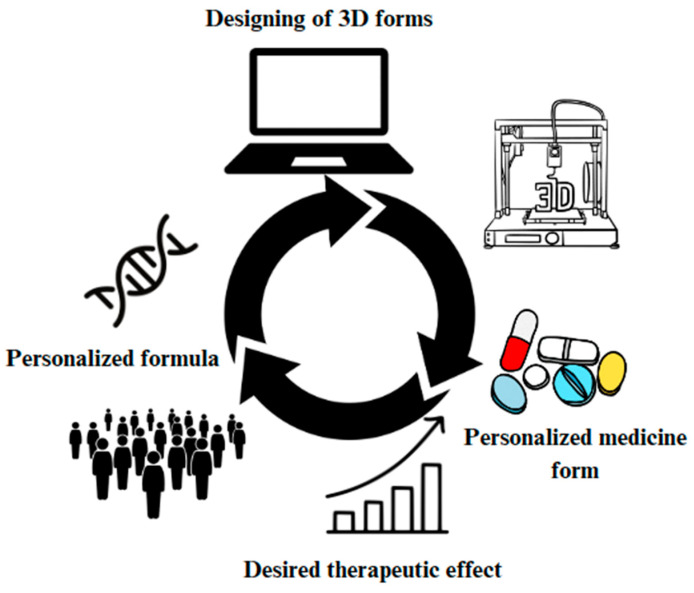
The cycle of personalized medicine development through 3D printing.

3D printing technology is a revolution in healthcare, enabling the development of precision dosing and individualized medication forms like self-nano-emulsifying drug delivery systems (SNEDDSs). Researchers have utilized this technology to create dosage forms with varied release profiles, enhancing the solubility and bioavailability of drugs such as dapagliflozin and glimepiride. Furthermore, this technology has been used to develop drug delivery devices like controlled-release shells [158,159], demonstrating the potential of 3D printing in personalizing medication regimens (Figure 8).

Historically, the unique physiological and metabolic requirements of children have been overlooked in pharmaceutical research, resulting in the need for pediatric-specific formulations. The pediatric population requires drugs that are not just dose-adjusted but are designed with a comprehensive understanding of pediatric pharmacokinetics. Manipulating adult formulations for children can lead to suboptimal efficacy and safety concerns [160]. Regulatory initiatives have underscored the importance of pediatric considerations in drug development, advocating for effective, palatable, and easy-to-administer medications for children [28,161,162].

The flexibility and personalization of 3DP offer numerous opportunities, especially in the development of medicines for the pediatric population [22]. It enables the generation of tailored doses (accounting for patients’ age, disease state, physiological function, and weight, eliminating the risk of over/under-dosing as a result of solid oral dosage form modification prior to administration), release profile, and design [163]. Children usually avoid taking tablets because they associate them with unpleasant sensations; chewable tablets may seem unpalatable, too hard, and tablets that must be swallowed often cause discomfort. Such unpleasant associations may encourage the child to avoid taking oral tablets. By using 3D printing it is possible to design the desired shape, size, and color tablet which would be more acceptable to children. Excipients play an important role as well. For example gelatin, due to its pliability, elasticity, and hydrophilicity, has been utilized as a 3D-printed biodegradable excipient in gummy antiepileptic drug formulations for pediatric patients via the extrusion base technique [14].

The geriatric population presents challenges, with age-related physiological changes affecting drug metabolism. There is a growing focus on research to adapt medication regimens to ensure safety and efficacy. The development of 3D-printed multi-drug tablets holds promise in simplifying medication schedules for older adults enhancing their treatment adherence [164,165]. The most important advantage for geriatric patients is 3D-printed oral dosage formulations (such as IDTs or ODTs) that are easy to swallow. The embossing designs, which indicate the time for administration for an individual person, are also suggested to be useful for patients with cognitive impairments and health assistants who must provide medication to these patients [14]. Other dosage formulations that are beneficial to geriatric patients are fast release tablets and sublingual tablets which provide the exact dose of APIs required. For patients who take multiple drugs at the same time, different drugs can be partitioned and combined into a single tablet to avoid errors or missed drugs, which can increase the safety and effectiveness of medication [13].

Furthermore, these multi-drug tablets extend beyond disease treatment to include personalized dietary supplements, suggesting broad applications in health management. Research has shown that while patients generally prefer conventional tablet shapes, they are open to selecting their medications’ color and design, indicating a preference for personalized treatment options [54,166].

However, there are challenges associated with 3D-printed medications, particularly concerning their physical characteristics. Studies have revealed that tablets with rough textures or unconventional shapes, such as pyramids or cuboctahedrons, can be challenging to swallow [167]. It is suggested that while technology allows for high customization, patient acceptability might depend heavily on the physical design of the tablets.

While multi-drug tablets present a promising route for more personalized and effective medical treatments, it is crucial to note that ongoing research is imperative.

**Figure 9:**
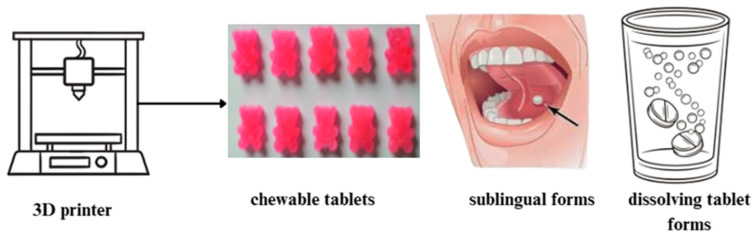
The various forms of medication, including chewable, sublingual, and dissolving tablets, created using a 3D printer for geriatric and pediatric patients [7,54,131].

However, innovations such as chewable tablets sublingual or dissolving tablet forms using 3D-printed technology [130,168] tailored to geriatric and pediatric patients are promising solutions (Figure 9).

These technologies allow for the customization of drug release profiles, dosages, and even flavors, making medications more accessible and palatable for elderly patients who may struggle with swallowing traditional pills or following complex medication schedules.

Several clinical studies have explored the use of 3D printing technology in pharmaceuticals, demonstrating its potential for personalized medicine. One significant study, conducted by FabRx, involved the world’s first clinical trial using personalized 3D-printed pills. This study focused on treating children with Maple Syrup Urine Disease (MSUD) by creating chewable tablets tailored to individual dosage needs using FabRx’s Printlets technology. The study found that these 3D-printed tablets were effective in controlling patients’ blood levels of isoleucine, showing better consistency and patient acceptability compared to conventional treatments [128].

Another study investigated the use of 3D printing in hospitals and pharmacies, highlighting its application in creating customized drug delivery systems such as oral controlled release tablets, implants, and microneedles [14]. The flexibility of 3D printing allows for precise dosing and the ability to produce medications with specific release profiles, which is particularly beneficial for patients with unique medical needs [14].

Additionally, the European 3D Printing Special Interest Group has been promoting clinical research to further investigate the use of 3D technologies in healthcare. These studies focus on developing patient-specific pills and other medical applications, with the goal of advancing personalized medicine and enhancing patient outcomes [169].

Three-dimensional printing is becoming more prevalent in personalized medicine, offering a promising approach to patient-centered care. This technique enables the creation of combinations of many drugs and tailored drug release patterns, specifically designed to meet the individual needs of children and geriatric patients. To fully take advantage of the potential of 3D printing in customized healthcare, it is crucial to continue performing research and implementing innovative solutions to overcome current obstacles as this sector continues to develop.

## 7. Three-Dimensional Pharmaceutical Printing for Patients with Chronic Diseases

Chronic diseases are defined as conditions that last 1 year or more and require constant medical attention or limit activities of daily living or both and could affect the patient’s quality of life. The most common chronic diseases primarily include Alzheimer’s disease, arthritis, asthma, cancer, diabetes, epilepsy, hypertension, obesity, and stroke [164]. Chronic diseases are associated with complex drug regimens as patients take lifelong medications to manage comorbid conditions in addition to the underlying disease (diabetes, cardiovascular disorders or cancer) that often lead to poor adherence to treatment. The use of extrusion-based 3D printing by combining various medications in the form of extended-release or sustained-release formulations, orodispersible tablets, and polypills form a great initiative for the treatment of multimorbid conditions [164]. Extended-release dosage forms offer the possibility of reducing the frequency of taking medicines in patients with chronic diseases. It also allows the combination of several APIs in a single tablet, thus reducing the incidence of side effects. The use of 3D printing technology contributes to avoid dosing errors and to improve adherence to the medication regimen, which is a prerequisite for achieving pharmacotherapeutic efficacy.

Diabetes mellitus (DM) is one of the common chronic diseases [170]. As the duration of DM and comorbid conditions increases, it is seen that patients need more than one drug for their glycemic control [170]. Polypharmacy is a common occurrence in the diabetic patient population and the application of 3D printing technology is one way to address this problem. Several research groups have made significant achievements in developing multi-drug tablets, offering a promising future for healthcare. For instance, Khaled and colleagues have initiated a tablet for hypertensive diabetic patients that contains three different drugs, each separated into different chambers to control their release rates [166]. This innovative approach simplifies the medication schedules of the patients and enhances compliance with prescribed treatments, potentially revolutionizing how to manage health conditions.

Three-dimensional printing not only enables the possibility to combine several APIs in one tablet but also the development of medicines containing unique doses of active ingredients for each patient. Triple oral therapy is a treatment regimen that employs a drug that is combined with two unsuccessful combinations of drugs in a polypill that results in a decrease in the glycemic index [164]. For example, 3D extrusion-based printing was used to produce polypills containing captopril, nifedipine, and glipizide with controlled release profiles [171]. These polypills can be used to treat hypertension, one of the co-morbidities of diabetes. However, some patients with diabetes suffer from other co-morbidities, such as atherosclerosis, dyslipidemia, chronic kidney disease or cardiovascular disease. For this reason, the dosage forms prescribed for each patient must be as personalized as possible.

To control atherosclerosis, 3D printing allows the combination of cholesterol-lowering pharmaceutical ingredients with anti-glycemic active ingredients [164]. However, for this form of medication to be effective, it is important to select the right excipients that have a sustaining effect. Hydroxypropyl methylcellulose (HPMC) and sodium carboxymethyl cellulose (CMC) are hydrophilic swellable polymers that are used to create sustaining layers. HPMC is a nonionic cellulose derivative that is widely implemented in the pharmaceutical field as a thickening agent for the drug matrix for controlled drug delivery [172]. Carboxymethyl cellulose (CMC) is derived from cellulose, which is a natural, biocompatible, biodegradable, and widely abundant biopolymer [173]. Polysorbate 80 and croscarmellose sodium are used to form an instant-release layer. The combination of sustained-release and instant-release layers in a tablet allows the modulation of the drug release rate according to the patient’s needs. FDM was also employed for the fabrication of a bilayer dosage form containing metformin and glimepiride, embedded in the Eudragit^®^ RL sustained-release layer and the polyvinyl alcohol (PVA) layer [164]. It is important to note that PVA is a biodegradable thermoplastic polymer and the use of a high polymer ratio in combination with the need for a high molecular weight of the polymers generates polymeric matrices with limited porosity, thus resulting in extended drug release patterns [44]. Eudragit^®^ RL polymer has high permeability and has been successfully used to create chronotherapeutic therapies based on pulsatile drug release.

Another area of focus for researchers is cardiovascular diseases. They are exploring the formulation of tablets that incorporate four or five drugs. These works highlight the vast potential of 3D printing in producing complex drug combinations tailored to individual patients’ unique needs [123]. Hypertension and dyslipidemia are modifiable risk factors associated with cardiovascular diseases (CVDs) and often require a complex therapeutic regimen [44]. Multiple and variable doses of drugs are prescribed for the patients to achieve a significant effect. For this reason, 3D-printing provides an opportunity to develop tablets with personalized doses of antihypertensive drugs for the cardiovascular patient population. As a result, flexibility can be achieved for this patient group. The administration of several medicines is commonly associated with poor levels of adherence among patients, to which the World Health Organization (WHO) proposed a fixed-dose combination unit (polypill) as a strategy to improve adherence [44]. However, printing polypills poses several technological challenges—when formulating such a dosage form, it is important to consider the potential for increased side-effects from combining APIs. Also, polypill needs to be individually tailored to each patient, taking into account the required dosage of each active substance, as well as the patient’s co-morbidities that can significantly impact the therapeutic effect of the drug. The polypill enables the possibility of incorporating an immediate release compartment and a sustained release compartment, thus facilitating the medication regimen for patients. As an example, a polypill was produced containing an immediate release compartment with aspirin and hydrochlorothiazide and three sustained release compartments containing pravastatin, atenolol, and ramipril [133]. It is important to consider the compatibility of active ingredients and excipients, as this may affect the stability of a medication. Another challenge in the formulation of a polypill is the different molecular structures of the APIs, which results in different physicochemical properties of each substance. Another important aspect to consider is the 3D printing method. Extrusion-based 3D printing methods and FDM have been applied to produce polypills. However, each of these methods has its own drawbacks when it comes to polypills. FDM 3D printing has been associated with a loss in drug content due to thermal degradation during hot melt extrusion (HME) and FDM 3D printing [44]. Extrusion-based methods require drying after printing, resulting in tablets with a fragile structure.

Cancer is another chronic disease that requires a personalized drug regimen. Although the standard treatment regimens for cancer are effective, systemic toxicity of chemotherapeutic agents and their continuous intravenous infusion can reduce patient compliance [174]. Oral medication is the most convenient form of administration and could lead to better adherence in cancer patients. However, the acidic gastric environment, microflora, and digestive enzymes lead to poor bioavailability of some drugs. Nanoparticles have been used as carriers of chemotherapeutic agents. The combination of nanotechnology and 3D printing was used to create a novel drug delivery system for colorectal cancer [174]. The application of 3D printing technology facilitates the adherence to the medication regimen which is necessary to ensure the effectiveness of pharmacotherapy in cancer patients. The 3D-printed tablets, with diameters of 10 mm and 13 mm, were produced using a drop-on-powder (DoP) or binder jetting method, which involved incorporating the anticancer drug 5-Fluorouracil (5-FU) [164]. Calcium sulfate hydrates, along with carbohydrates and vinyl polymer, were used as the base powder to create the structure of the tablet. 2-Pyrrolidone was chosen as the water-based binding liquid, functioning as the inkjet ink. The tablet surfaces were coated with polymers such as Soluplus (SOL) and Polyethylene Glycol (PEG), that enable the controlled release of the drug. These coatings enhance drug solubility and regulate the release of 5-FU during treatment. Soluplus^®^ is a novel solubilizer and a matrix-forming polymer. This solubilizer is ideal for APIs that are poorly soluble and can be used in the HME 3D printing method as it shows no chemical degradation, even after extrusion at 220 °C. However, binder jetting has benefits such as easy adaptation and fixation, reduced development time, and favorable aesthetic results [175].

Printing personalized dosage forms helps to design medicines according to the individual health characteristics of each patient with a chronic disease. It also enables the optimization and simplification of the medication regimen. These factors contribute to solving polypharmacy problems, which are particularly common in the chronic disease patient population.

## 8. Novel Dosage Forms Developed Using 3D Printing: Future and Innovation

Three-dimensional printing technology has enabled the development of innovative pharmaceutical dosage forms with the aim to enhance patient compliance, improve drug efficacy, and offer new mechanisms for drug delivery, such as topical patches, orodispersible forms, dissolving oral films, implantable devices, or multilayer pills [9,13,15]. Three-dimensional printing enables the production of individualized medications that are customized to meet the unique requirements of each patient, including the exact dose, release profiles, and drug combinations [14,20]. This could be particularly beneficial in the cases where the unique dosages that are not available in the standard pill sizes are required, or multiple medications are prescribed that could be combined in a single pill containing all of them [14,20]. With 3D printing, it is possible to create complex structures within a tablet that control how the active ingredient is released over time [9,13,14,20]. This can be used to create medications with delayed, sustained, or sequential release, which can help to improve treatment effectiveness [9,13,14,20]. Porous matrices or other novel structures that can enhance the solubility and absorption of drugs within the body could be produced by 3D pharmaceutical printing, thus solving the poor water solubility problems of particular drugs [176].

Three-dimensional printing can produce customized drug-loaded patches for dermatological application, allowing for the controlled release of medications [177,178]. These patches can be designed to conform to the unique contours of the application area on the body of the patient, ensuring better contact and effectiveness; also, they could be adapted to deliver antibiotics or anti-inflammatory medications directly to the affected skin area, optimizing therapeutic outcomes while minimizing systemic side effects [177,178].

Three-dimensional-printed microneedles offer precise, minimally invasive drug delivery across the skin barrier, enhancing bioavailability and consistency compared to oral or injectable routes [179,180,181,182]. They can be engineered to overcome poor skin permeability, improving therapeutic efficacy [183,184]. Customizable in drug penetration and release, microneedles have shown success in delivering analgesics, epilepsy treatments, and Alzheimer’s medications with sustained release, effective skin permeation, and biocompatibility [179,180,181,182]. An analgesic microneedle patch has been developed using dissolvable microneedles to transdermally deliver a selective calcitonin gene related peptide, a neuropeptide released from sensory nerve endings [185]. Donepezil-coated microneedle patches for Alzheimer’s treatment, produced using DLP and SSE 3D printing, demonstrated effective drug incorporation, strong mechanical properties, enhanced skin permeation, sustained release, and biocompatibility, highlighting their potential for clinical transdermal application [186]. Three-dimensional-printed microneedles enable multi-drug delivery, unlike conventional patches, and can integrate diagnostics with therapy, such as colorimetric sensors for real-time health monitoring and on-demand drug release [187]. Personalization is another advantage, allowing tailored needle lengths for improved drug absorption in patients with skin conditions like diabetes or psoriasis [188].

Three-dimensional printing is especially useful in producing orally disintegrating tablets [13,146,152] and modified release oral dosage forms [13,189,190]. “Spritam” by Aprecia Pharmaceuticals was the first FDA-approved drug produced by 3D printing [191]. It utilizes ZipDose Technology, which creates a porous formulation that dissolves rapidly with a sip of liquid, making it easier to take [191]. Three-dimensional printing could be used to produce fast dissolving oral films, thin strips that dissolve quickly upon contact with saliva, delivering active pharmaceutical ingredients directly to the bloodstream via the oral mucosa [192]. Using 3D printing, these films can be customized to contain precise dosages customized according to the needs of the patients [192]. They are especially beneficial for pediatric and geriatric populations who may have trouble swallowing pills [192,193]. The films can carry a range of medications, from pain relievers to antihistamines, and can be flavored to make the medicine more palatable [192,193].

Three-dimensional printing is used to create personalized implantable devices that can release drugs at a controlled rate directly to the target area within the body [12,194,195,196]. These properties are important in treatments requiring localized drug delivery over extended periods, such as chemotherapy agents or antibiotics [12,195,196]. An example is implants used in periodontal therapy, where 3D-printed biodegradable frames slowly release antibiotics to treat infections [12,195,196].

Multi-layered pills where each layer contains a different drug, releasing each active ingredient at different times or rates, could be produced by 3D pharmaceutical printing [73,133,155,197,198]. This technology simplifies complex medication schedules, significantly enhancing adherence [133]. This method is particularly beneficial for patients with chronic conditions such as diabetes and hypertension, who often need to take several medications daily [155,197,198]. An innovative example might be a cardiovascular polypill that combines blood pressure medication into a single, once-daily pill tailored to individual patient needs [198].

In remote or under-resourced settings, 3D printers could be used to manufacture medications on-site, reducing the need for large-scale production facilities and logistics [13,14,193]. For example, in response to the COVID-19 pandemic, some hospitals used 3D printing to create on-demand batches of nasopharyngeal swabs and other critical supplies [199]. Extending this approach to pharmaceuticals, 3D printers could be used in remote areas to produce drugs as needed, reducing the need for large stockpiles and improving access to essential medications [13,14,193].

Moreover, rapid prototyping of tablets can facilitate clinical research by allowing quick adjustments to dosages and formulations based on early trial results [14,200]. The drug delivery system could be changed to immediately test modifications, streamlining the development process and potentially bringing effective treatments to the market faster [14,200]. This flexibility could significantly reduce the time and cost associated with drug development [13,14,200].

These examples highlight the versatility and potential of 3D printing in creating more effective and patient-friendly pharmaceutical dosage forms. This technology not only supports the development of novel drug delivery mechanisms but also opens new possibilities for personalizing medicine to meet individual therapeutic needs.

## 9. Overview of the Specifications and Requirements of 3D Printing in the Pharmaceutical Industry

The implementation of 3D printing in the pharmaceutical industry involves specific technical specifications and regulatory requirements to ensure safety, efficacy, and quality [9,13,14,15]. Technical specifications for 3D printing for pharmaceutical applications present the precise requirements and standards that must be fulfilled during the design and setup of 3D printers used to make pharmaceuticals [9,13,14,15]. These specifications include the types of printers (such as inkjet or fused deposition modeling), the materials used (such as pharmaceutical-grade polymers and biocompatible resins), the resolution and precision of the devices, and the software needed to design drug formulations with specific dose and release characteristics [9,13,14,15]. Furthermore, these requirements include essential factors related to the stability, efficacy, and safety of printed pharmaceutical items, ensuring that they meet the high regulatory standards established by health authorities [9,13,14,15,201]. The precise elaboration of technical specifications is critical for attaining the desired therapeutic outcomes, maintaining quality control, and guaranteeing that each batch of printed medication is consistent with its design and safe for patient use [9,13,14,15,201].

Three-dimensional pharmaceutical printers must have high resolution and precision to accurately produce complex structures with controlled porosity and surface area [9,21,176]. This is crucial for managing drug release rates and dose accuracy. Moreover, 3D printers must be compatible with pharmaceutical-grade materials, including polymers and active pharmaceutical ingredients, that meet safety and efficacy standards [9,12,14]. These materials should not react with the drug or alter its properties [9,12,14]. Equipment must maintain sterility and prevent contamination during the printing process, including the use of sterile materials and maintaining a clean production environment [9,14].

While 3D printing is ideal for small-scale production and prototyping, expanding to larger quantities can be challenging [14,202]. The technology needs to be capable of efficient scaling without compromising product quality. Furthermore, speed is often a limiting step in 3D printing. Pharmaceutical applications require a balance between print speed and the complex requirements of drug formulations, including the accurate dosing of multiple ingredients [14,202].

Technical specifications outline the abilities and limitations of printers, materials, and processes, ensuring that each batch of medication not only satisfies strict health standards but also delivers the desired therapeutic results [14,202]. Precision in design, material integrity, and process control are essential for creating safe and effective drugs [9,13,15,18]. As the field of 3D pharmaceutical printing evolves, it will be critical to refine these technical criteria in order to take full advantage of the 3D printing, which will eventually lead to improved patient-specific treatments and drug delivery innovations [9,13,15,18].

Each batch of printed medications must undergo rigorous testing to ensure consistency and quality [14,17,201]. This includes validation of the printing process and verification of the drug release profiles, dosage accuracy, and stability [14,17,201]. Production facilities must comply with GMP standards, which include requirements for quality management, personnel qualifications, cleanliness, equipment verification, and process validation [14,17,201]. Since 3D printing often involves the digital transmission of formula and design files, securing the data against unauthorized access and ensuring integrity throughout the process is crucial [14,17,201]. It is also essential to maintain detailed records of the manufacturing process, materials used, and testing results for each batch. This traceability aids in addressing any quality issues and managing recalls if necessary [14,17,201].

Numerous studies and patent applications have focused on medical and pharmacological applications of 3D printing. The “Technical Considerations for Additive Manufactured Medical Devices” guidance was released in 2017 by the United States Food and Drug Administration (FDA) [201]. This document summarizes the FDA’s recommendations from device development to process validation and acceptance activities, including many aspects of device design, manufacture, testing, and labeling. This guidance is also applicable for 3D printing use in hospitals, medical centers, or pharmacies. In December 2021, a discussion paper was issued with the plans of the FDA to develop draft and final guideline materials depending on feedback. There have been proposals for the FDA to adopt standards or guidelines outlining how it assesses device risk and regulates products accordingly [201]. However, the FDA had not yet adopted explicit recommendations or restrictions for employing 3D printing in the production of pharmaceuticals [14,201].

In Europe, the European Medicines Agency (EMA) also plays a crucial role in setting guidelines and standards for this fast-developing sector. Like the FDA, the EMA has not yet established regulations specific to 3D-printed drug forms [203]. Manufacturers using 3D printers for medical devices and drug production must perform risk assessments to meet the health and safety standards set out in the Machinery Directive 2006/42/EC [204]. However, the EU legal framework is designed to be technologically neutral, not requiring specific technical solutions for product design, thereby allowing manufacturers to adopt various technical approaches to meet these critical standards [14].

Future developments could integrate 3D-printed pharmaceuticals with digital health technologies, such as smart pills and digital tracking, to further enhance patient outcomes and compliance. The sustainability of the manufacturing process and the recyclability of materials used in 3D printing should also be considered. Balancing the cost of advanced 3D printers and materials with the need to make healthcare accessible and affordable remains a significant challenge to be tackled in the future [9,13,14,15]. Clear guidance documents on the use of 3D pharmaceutical printing should be provided to proceed with the rapid development of this important innovation and its application for the disease control and management and the benefits for the patients and medical care specialists.

## 10. Production of Customized 3D-Printed Pharmaceutical Dosage Forms

The production of customized 3D-printed pharmaceutical dosage forms is a process that begins with the design and formulation process, where pharmacists or scientists determine the specific characteristics of the medication tailored to individual patient needs, including the dosage, release mechanism, and physical design. A precise amount of active pharmaceutical ingredient is chosen based on the patient’s condition, age, weight, and other clinical factors. Optimal release characteristics are achieved by designing the structure of the pill to control the release of the medication, such as immediate, delayed, or sustained release [12,16,17,20]. Customizing the shape and size of the pill helps to enhance patient compliance, which is especially useful in pediatric and geriatric populations where swallowing large pills can be difficult [22,23]. The design is created by using sophisticated software that generates a digital blueprint of the medication [12,16,17,20]. Materials for the 3D printing process are carefully selected based on compatibility with the active pharmaceutical ingredients and desired properties such as solubility and stability. Common materials include pharmaceutical-grade polymers and excipients. The choice of material can enhance the absorption of the drug into the body and ensure that the drug remains effective until its expiration date. Materials must not react with active pharmaceutical ingredients or alter their therapeutic effects [12,16,17,20].

Using the blueprint, the medication is then produced with a 3D printer, which could employ techniques such as powder bed printing, extrusion printing, or inkjet printing to construct the dosage form layer by layer. The precision of this technology allows for complex structures that can control how the medication is released into the body. After printing, the dosage forms often undergo various post-processing steps like drying, curing, or coating to enhance their stability and effectiveness [13,15,193]. Quality control is crucial, with each batch tested for uniformity, dissolution rates, and mechanical strength to ensure it meets strict pharmaceutical standards [14]. Finally, the finished products are packaged with specific labeling to protect them from environmental factors and provide essential patient information before being distributed. This entire process is regulated stringently to ensure each customized medication is safe and effective for patient use [14,17].

This innovative approach not only enables more precise and personalized medication therapies but also has the potential to improve patient compliance and overall treatment outcomes, marking a significant advancement in pharmaceutical manufacturing [14,17].

## 11. Artificial Intelligence (AI) in 3D Pharmaceutical Printing

The integration of artificial intelligence (AI) in the pharmaceutical industry has progressed remarkably over the past few decades, revolutionizing drug discovery, development, and personalized medicine [205,206]. In the 1980s and 1990s, AI applications were primarily limited to computational modeling for molecular structure prediction. These early foundations paved the way for more advanced machine learning techniques in the early 2000s, enabling the analysis of complex datasets to predict molecular interactions and optimize drug formulations [205,206]. By the 2010s, the widespread adoption of AI was accelerated by advances in deep learning, big data, and access to extensive biological datasets from genomics, proteomics, and high-throughput screening. Pharmaceutical companies began leveraging AI across various stages of drug development, from target identification and formulation design to clinical trial optimization [205,206].

AI-driven drug discovery has significantly facilitated the identification of novel therapeutic targets, reducing reliance on traditional, time-consuming experimental methods. It has also played a crucial role in drug repurposing by identifying new therapeutic applications for existing drugs, expediting their transition from laboratory research to clinical use [205,206]. This is particularly beneficial for treating rare and neglected diseases where conventional drug development is often impractical.

The integration of AI in 3D drug printing could improve drug design, formulation optimization, and production efficiency [207]. AI-driven algorithms analyze vast datasets to tailor drug compositions, dosage forms, and release profiles according to individual patient needs [208], significantly improving treatment efficacy [205,206]. AI enhances computer-aided drug design by predicting the optimal material properties, excipient combinations, and structural configurations for 3D-printed pharmaceuticals [209]. Machine learning models can simulate and optimize drug dissolution rates, bioavailability, and stability, reducing the need for extensive trial-and-error testing [210]. Deep learning techniques help refine printing parameters, such as temperature, extrusion speed, and layer thickness, ensuring consistent drug quality and minimizing production errors [205,206]. AI also improves real-time monitoring and quality control in 3D drug printing. Additionally, AI-driven generative design facilitates the creation of complex drug geometries, such as multi-layer tablets with controlled-release mechanisms, optimizing drug delivery and absorption rates [208,209]. The integration of 3D printing and AI in microneedle fabrication was recently explored, highlighting the role of AI in predicting drug release patterns, enhancing quality control, and enabling autonomous manufacturing through machine learning and the internet of things, and their role in biosensing applications [211].

In clinical settings, AI-powered personalized medicine platforms analyze patient data, including genetic profiles, metabolic rates, and medical history, to generate customized drug formulations. This allows for precision dosing, reducing adverse effects and improving therapeutic outcomes [205,206].

Despite its transformative potential, AI adoption in pharmaceuticals faces critical challenges, including ethical considerations, regulatory constraints, data privacy concerns, and the need for robust validation methodologies 181,182]. Regulatory agencies, such as the FDA and EMA, are actively working to establish clear guidelines to ensure AI-driven drug discovery and 3D-printed pharmaceuticals meet safety, efficacy, and quality standards. The continuous refinement of AI algorithms requires rigorous validation against independent datasets to maintain model accuracy and prevent biases, particularly in handling imbalanced datasets. Techniques such as cross-validation, ensemble modeling, and precision–recall analysis help mitigate risks associated with overfitting and unreliable predictions [205,206].

In January 2025, the FDA released draft guidance titled “Considerations for the Use of Artificial Intelligence To Support Regulatory Decision-Making for Drug and Biological Products” [212]. This document offers recommendations for the industry on utilizing AI to generate data intended to support regulatory decisions concerning the safety, efficacy, or quality of drugs.

In December 2023, the EMA endorsed a multi-annual workplan to guide the integration of AI into medicines regulation across Europe [213]. This plan emphasizes four key areas: providing guidance and policy support for AI applications throughout the medicinal product lifecycle, developing frameworks for AI tools and technologies, fostering collaboration and training to build AI capacity, and promoting structured experimentation with AI solutions [213]. Additionally, the EMA released a reflection paper detailing considerations for the use of AI and machine learning in drug development, covering stages from discovery and non-clinical development to clinical trials and post-authorization activities [214]. This document encourages a risk-based approach to AI tool development and deployment, ensuring that AI applications are both safe and effective.

As AI becomes an indispensable tool in pharmaceutical research, ongoing collaboration among researchers, clinicians, industry stakeholders, and regulatory bodies is essential for navigating these challenges. The future of AI in drug development promises increased efficiency, reduced costs, and groundbreaking innovations in precision medicine, ultimately transforming the way pharmaceutical treatments are designed, tested, and delivered.

## 12. Future Directions and Perspectives

The future of 3D printing in the pharmaceutical sector promises exciting developments and significant changes in how pharmaceuticals are developed, produced, and distributed [13,215]. Future developments will likely include more advanced 3D printers that can handle a wider variety of pharmaceutical materials with higher precision and at lower costs. Research into new materials compatible with 3D printing technologies will expand the range of possible drug formulations, allowing for more active pharmaceutical ingredients and excipients to be used. Technological innovations might enable the simultaneous printing of multiple drugs with distinct release profiles within a single dosage form. This would be particularly beneficial for patients managing multiple chronic conditions, simplifying their medication regimens [9,15].

As 3D printing technology develops very fast, regulatory bodies like the FDA may consider more streamlined pathways for approving 3D-printed medications. This could involve creating frameworks that can adapt to the high variability and customization potential of 3D-printed drugs, possibly through adaptive licensing approaches [201]. New standards and guidelines should be established to ensure the safety, efficacy, and quality of 3D-printed pharmaceuticals. These guidelines should address aspects such as manufacturing consistency, drug stability, and patient safety [201].

As the technology becomes more accessible and cost-effective, 3D printing could be routinely used in hospitals and clinics for on-demand manufacturing of drugs, especially for urgent, rare, or discontinued medications [14]. The ability to print drugs in pharmacies could revolutionize treatment strategies, especially in remote or underserved locations [14]. This approach would reduce logistics costs and improve access to personalized medicine.

There probably will be ethical considerations regarding access to 3D-printed medications, including how to ensure that all patients can benefit from this technology regardless of socioeconomic status. It could also lead to a wide discussion about intellectual property rights concerning drug formulas and the potential for their misuse [9,13,193]. Addressing disparities in healthcare access will be crucial, as 3D printing has the potential to either narrow or widen the gap between different population groups depending on its implementation [9,13,193].

Three-dimensional printing could facilitate the development of more complex drug delivery systems, such as those capable of targeting specific tissues or cells, or responsive systems that activate under certain physiological conditions. Continued focus on personalized medicine could speed up innovations in 3D printing, enabling even more precise customization of drug therapy to individual genetic profiles, lifestyles, and health conditions [9,13,193].

However, the widespread adoption of 3D printing in the pharmaceutical industry faces several critical challenges that must be addressed [6,202]. The successful integration of 3D printing into mainstream pharmaceutical manufacturing necessitates unprecedented collaboration between industry stakeholders, regulatory bodies, and academic researchers. One of the primary disadvantages of 3D printing technology in pharmaceuticals is the lack of regulatory guidelines and standardization [78]. Currently, no clear regulatory frameworks or guidelines govern the use of 3D printing to manufacture pharmaceutical products. This lack of regulatory guidance poses a significant barrier to the widespread adoption of this technology, as pharmaceutical companies and healthcare providers require regulatory certainty to ensure the safety and efficacy of 3D-printed drugs [14,216].

Another critical challenge is the technical limitations of 3D printing technology. Some 3D printing techniques, such as fused deposition modelling (FDM), may not be suitable for fabricating thermolabile APIs due to the high temperatures. (Table 1) Additionally, the accuracy and precision of 3D printing can be affected by various parameters, such as the viscosity of the printing material, the nozzle size, and the printing speed, which must be carefully controlled to ensure the consistent production of high-quality drug products [13]. Often, the available materials are not optimal for all types of drugs, particularly those that are highly sensitive to environmental conditions or require very precise delivery mechanisms [217,218]. Moreover, the resolution of 3D printers can limit the minimization of doses, which is critical for delivering precise amounts of highly potent APIs. This limitation can lead to variability in drug release rates, potentially affecting the efficacy and safety of the medication [219,220]. Further research and development are needed to enhance printer resolutions and develop new materials that are compatible with a broader range of pharmaceutical applications.

Furthermore, the scalability of 3D printing in the pharmaceutical industry is a significant concern. While 3D printing offers the ability to personalize drug products, more than the throughput of current 3D printing technologies may be required to meet the demands of large-scale pharmaceutical manufacturing. To address these challenges, the pharmaceutical industry, regulatory agencies, and research institutions must collaborate to develop new standards, guidelines, and innovative 3D printing techniques that can overcome technical limitations and enable the scalable production of 3D-printed drugs [14,75,221].

Future research in pharmaceutical and biomedical 3D printing should emphasize the development of innovative, biocompatible, and biodegradable materials that offer precise control over drug-release profiles, stability, mechanical properties, and patient compatibility. Enhanced printing techniques with reliable, reproducible precision for multi-drug formulations, incorporating customized release kinetics and integrated diagnostic sensors, will be critical for expanding therapeutic efficacy, personalized medicine applications, and patient-specific treatments. Comprehensive regulatory frameworks, clear guidelines, and standardized quality controls must be established to facilitate the validation, approval, and commercialization of 3D-printed dosage forms. Advancing personalized medicine through automated 3D printing will allow tailored therapies and devices such as microneedle arrays, responsive-release implants, and stimuli-triggered systems. Additionally, overcoming technical and economic barriers to scalability and commercialization is vital to ensure practical implementation, broad accessibility, and clinical integration of advanced 3D-printing technologies.

Thus, 3D pharmaceutical printing has the potential to increase customization, accessibility, and capability in drug formulation and delivery. As this field evolves, it will likely lead to significant changes in both the technical and regulatory landscapes of pharmaceutical manufacturing, with a particular focus on improving patient outcomes and optimizing healthcare delivery.

## Figures and Tables

**Table 1:** Comparison of 3D printing technologies for pharmaceuticals.

Technology	Mechanism	Advantages	Disadvantages
**FDM (fused** **deposition** **model)**	FDM operates by heating a drug-infused polymer filament and extruding it through a nozzle that follows computer-controlled paths, depositing the material layer by layer onto a build platform where it solidifies upon contact. This process allows for the creation complex dosage forms through precise, layer-by-layer fabrication [30].	Simple setup, economical, wide range of materials, ability to create complex dosage forms [45,46].	Potential drug degradation due to high temperatures, lower resolution, coarser surface finish, and structural weaknesses at layer junctions [78,79].
**DPE (direct powder extrusion)**	DPE combines drug powders with binders and extrudes and fuses them into solid forms without solvent [55].	Without solvent, high drug load, reduced post-processing [55].	Powder flow issues, limited excipient compatibility [55].
**SSE (semi-solid extrusion)**	SSE involves extruding semi-solid materials, such as gels or pastes, from a syringe-like system in successive layers to form a 3D object. The materials are carefully blended to achieve the ideal viscosity for printing, allowing for the creation of complex drug formulations at lower temperatures to preserve the integrity of the active ingredients [59].	Versatile, suitable for temperature-sensitive drugs, low waste generation, and efficient [61].	Requires careful selection of excipients, the potential for shrinkage and deformation during post-processing, and lower resolution prints [61].
**SLS (selective laser sintering)**	SLS uses a laser to selectively fuse powder particles on a powder bed according to a predetermined pattern. After each layer is fused, a new layer of powder is spread over the bed, and the process is repeated layer by layer to construct the final 3D object [63].	High precision, no need for support structures, suitable for complex drug delivery systems [64,65].	High equipment costs and intense laser energy can cause drug degradation and challenge scaling up large production [64,65].
**BJ (binder jetting)**	Binder jet printing involves a nozzle that dispenses a binding liquid onto a flat powder bed, moving along the *x*–*y*-axis. This liquid binds the powder particles together, solidifying each layer. The build plate then lowers along the *z*-axis, and a new powder layer is applied, with the process repeating layer by layer to create the final 3D-printed structure [69].	High-resolution parts are versatile and suitable for complex and multi-drug formulations [69,70].	It requires post-processing for strength, a less smooth surface finish, and is limited by binder droplet size and powder granularity [1].
**SLA (stereolithography)**	SLA involves exposing a photopolymer resin to a high-energy light source, such as UV light, which induces polymerization and solidification of the resin. The platform moves down vertically along the *z*-axis after each layer is solidified, and the process repeats with a new layer of resin being applied and cured, building the 3D object layer by layer [73,74].	High precision, cost-effective, suitable for complex drug delivery systems, and minimal local heating preserves heat-sensitive drugs [34,75].	Limited photocrosslinkable polymers are approved for medical use, there are potential safety concerns with some resins, and the stability of drug formulations is compromised [34,75].

**Table 2:** Innovative excipients used for the development of chewable tablets.

Materials	Functions	References	3D Printing Techniques
Gelucire 48/16 Klucel ELF	Excipients	[19]	FDM, SSE
Xanthan gum	Excipients	[20,21]	SSE
Carrageenan-gelatin	Excipients	[22]	SSE
Gelatin	Excipients	[21,23,24]	SSE
Bitter chocolate	Excipients	[25]	SSE
Corn (glucose) syrup Potato starches	Excipients	[14]	SSE

**Table 3:** Excipients used for the development of IDTs and sublingual pharmaceutical forms [11,144,145].

Materials	Functions	References	3D Printing Techniques
Copovidone VA 64, Mannitol	Excipients	[146,147]	SLS
HPβCD (72.1%) HPMC F4M (1.4%) NaCCS (2.5%)	Excipients	[148]	SSE
HPMC E5	Excipients	[149]	SLS
Polyvinylpyrrolidone (PVP) PEO and PVA	Excipients	[145]	SSE
Maltodextrin, HEC Cellosize^®^, Sorbitol (plasticizer)	Excipients	[150]	FDM
HPMC and glycerol	Excipients	[151]	IJ
HPMC E5 and Kollidon^®^ VA 64	Excipients	[152]	SLS
MCC PH 101 Peorlitol 50C Aerosil 200	Excipients	[139]	BJ-3DP
PEG 6000 HPC Sodium starch glycolate Croscarmellose	Excipients	[54]	FDM
Kollidon VA 64 Kollicoat IR Mannogem XL Compressol SM	Excipients	[153]	FDM

## Data Availability

All data are available upon request.
